# Can Communication Strategies Combat COVID-19 Vaccine Hesitancy with Trade-Off between Public Service Messages and Public Skepticism? Experimental Evidence from Pakistan

**DOI:** 10.3390/vaccines9070757

**Published:** 2021-07-07

**Authors:** Qiang Jin, Syed Hassan Raza, Muhammad Yousaf, Umer Zaman, Jenny Marisa Lim Dao Siang

**Affiliations:** 1 Intercultural Communication Research Center, Hebei University, Baoding 071000, China; jinqiang@hbu.cn; 2 Department of Communication Studies, Bahauddin Zakariya University, Multan 66000, Pakistan; hassansherazi@bzu.edu.pk; 3 Centre for Media and Communication Studies, University of Gujrat, Gujrat 50700, Pakistan; m.yousaf@uog.edu.pk; 4 Endicott College of International Studies, Woosong University, Daejeon 34606, Korea; 5 Department of Business Administration, School of Management, National Central University, Taoyuan City 32001, Taiwan; 109481606@cc.ncu.edu.tw

**Keywords:** COVID-19 vaccine, vaccine hesitancy, public service message, health belief model, affect theory, public skepticism, willingness to take COVID-19 vaccine

## Abstract

The COVID-19 pandemic may have reached a turning point as the World Health Organization and the global community of nations step up plans for mass vaccination campaigns. However, the COVID-19 vaccine-related conspiracy theories (e.g., falsehoods about birth control, women infertility, surveillance, and microchip humanity, etc.) have built new momentum for vaccine hesitancy. To this end, several nations worldwide, including Pakistan, are struggling to boost public trust and enthusiasm to get vaccinated, especially in an anxious and complicated atmosphere propelled by multiple, new and the deadliest variants of COVID-19. To address this critical research gap during these intensifying conditions of vaccine hesitancy, the present study makes the first attempt to explore the potential effects of various communication strategies, including public service message (safety benefits versus fear appraisals), media types (i.e., traditional versus digital), self-efficacy, perceived benefits and threats (susceptibility and severity), on the willingness to get vaccinated for COVID-19. Importantly, the underlying effects of public skepticism (in a moderating role) on these relationships were empirically examined. Using four fictitious COVID-19 immunization campaigns in a series of experiments with 2 (media type: traditional vs. digital) X 2 (service attribute: health and safety benefits vs. fear) message frames (represented as Group one to Group four), the findings identified fear appraisal as the most viable communication strategy in combating vaccine hesitancy. Moreover, public skepticism negatively moderated the effects of media types and public service message attributes on willingness to get vaccinated in relatively high (i.e., Group two), moderate (i.e., Group one and four), and low intensities (i.e., Group three). The pioneering findings of this research offer new strategic insights for the global health authorities and vaccine promoters to proactively address the downward spiral of people’s willingness to take the COVID-19 vaccine.

## 1. Introduction

The global transmission of the new “Delta COVID-19” variant has edged a new race against the COVID-19 vaccines, hence creating a major threat to vulnerable populations with lower vaccination rates. The coronavirus (COVID-19) is caused by the severe acute respiratory syndrome coronavirus 2 (SARS-Cov-2) [1]. It was first reported in Wuhan, China, in December 2019 [2,3,4]. The disease then spread quickly across the globe, causing severe pressure on governments and regional economies, in addition to challenging healthcare workers and facilities and the general public [5]. The unprecedented and rapid spread of the COVID-19 pandemic made it the century’s most prevalent public health emergency [6]. Thus, on 11 March 2020, the World Health Organization (WHO) declared COVID-19 as a pandemic. Apart from promoting awareness about the precautionary measures, the development of a safe and efficacious COVID-19 vaccine has been a foremost obligation in combating COVID-19 [7]. Vaccination against COVID-19 is essential to control and prevent COVID-19 [8]. To accomplish this, about 70 percent of the population needs antibodies, either through contracting the virus or through inoculation [9]. As of January 2021, numerous vaccines have largely claimed to be safe and efficacious in producing protection against COVID-19. The provisional results of stage three trials of competing COVID-19 vaccines revealed higher efficacies between 80–95% [7]. Many of these vaccines have now been approved for public usage across the globe, leading to a mass vaccination global campaign (i.e., an operative deterrent) against the COVID-19 pandemic [10]. 

Albeit COVID-19 vaccines have been verified to be safe for human health and efficacious against COVID-19, but their widespread acceptance is a worrying issue, particularly in developing nations [11]. For example, with the beginning of vaccination campaigns in some nations, also came an extensive dissemination of fake information about vaccination usage on social media platforms that widely affected the acceptance of COVID-19 vaccines [3]. This exposure to online misinformation significantly increases susceptibility that induces a fall in COVID-19 vaccination intent [7]. Likewise, misinformation and other factors (e.g., skepticism about vaccine development) contribute to vaccine hesitancy across the globe [12,13]. Moreover, owing to safety concerns, there is a large public divide on getting vaccinated [10,14]. In this vein, various conspiracy theories (e.g., coronavirus is bioengineered, linked to implanting microchips, population control, and cause infertility, etc.) have widely spread into mainstream and social media that are negatively related to the vaccination uptake [15,16,17]. For instance, recent studies have found that 14–30 percent of the general public is hesitant to get vaccinated [18,19,20,21,22]. Owing to these discourses, vaccine hesitancy is still a severe issue to the effort to control the spread of COVID-19 [18]. 

However, it is noted that threat appraisal corresponds to an increase in vaccination willingness [7]. Similarly, it was found that participants who have a higher perceived severity of COVID-19 infection, perceived susceptibility, and perceived benefits of COVID-19 vaccination were more willing to take the COVID-19 vaccine [23]. To increase willingness and minimize hesitancy among the public to get vaccines requires publicity of the social benefits of the vaccines [24]. Furthermore, recent research on COVID-19 has remained limited in addressing the critical issues by providing a strategic and communicative solution. Most of the recently published work offers descriptions about the beliefs that contribute to the dearth of motivation among the general public to take the COVID-19 vaccine [21,23,25,26]. These recent studies on COVID-19 have overlooked the cognitive and emotive mechanisms of how individuals process health information, instead emphasizing media content analysis [3,16,17]. Concerning the information processing model and persuasive health messages as interpreted by the audience, this study specifically articulates the cognitive and emotive mechanisms [16,17]. This study clarifies the role of fear and safety appraisals in public service messages, primarily in COVID-19 vaccination awareness campaigns. Importantly, this research provides experimental evidence to address some of the most intriguing and timely questions, i.e., (a) how individuals process COVID-19 related public service messages; (b) how their cognitive, affective, and behavioral reactions to the COVID-19 related public service messages are affected by the message attributes, and (c) how these responses are affected by the medium of the message. There is no prior study documenting the interplay between health risks, safety recommendations, and media selection [3,16]. However, to our knowledge, this is the first study that identifies the most effective source and message attributes for scheming the COVID-19 vaccination awareness campaigns [11,26,27].

Thereby, this study takes a novel approach to COVID-19 vaccine acceptance as it sets out to deliver strategic and timely information toward the ongoing vaccine hesitancy problem. The critical underpinning question is about the role of public service messages’ strategic design in developing people’s motivation to get vaccinated. Therefore, the Health Belief Model (here-after HBM) serves as a theoretical underpinning in ascertaining vital constructs to investigate the subsequent COVID-19 vaccine-related beliefs: perceived susceptibility (susceptible to COVID-19), perceived severity (how risky is COVID-19), perceived benefits (pros of COVID-19 vaccine immunization such as health benefits, etc.), perceived barriers (skepticism towards COVID-19 vaccine: population control, etc.), and self-efficacy. This research initially recognizes the benefits, barriers, and threats of COVID-19 from the literature, then defines the cues to action, including disclosure to a persuasive message via media contents such as public service messages (a form of advertising), to promote willingness to take the COVID-19 vaccine [11,26,27]. Recent studies underpinning the HBM suggest that the efficacious administration of COVID-19 relies on the effective dissemination of factual information by activating cognitive mechanisms [28]. Albeit the HBM includes critical facades of cognitive mechanisms, but it has not sufficiently explained the emotions involved in inducing preventive behaviors [29]. However, the affect theory of persuasion diversified the existing body of knowledge by highlighting the role of emotional factors. Consequently, the current study fills the gap by incorporating the HBM and affect theory to experiment with the efficacy of the public service message frames, including fear appraisal (i.e., emotions) and safety benefits (i.e., cognitive).

For this reason, the study mainly designed four COVID-19 vaccination awareness campaigns to comparatively examine the competencies of fear appraisal (emotions) and safety benefits (cognitive) across traditional and digital media. This experimental study, therefore, investigates the effect of self-efficacy, perceived susceptibility, and perceived benefits along with action cues and barriers on the willingness to take the COVID-19 vaccine. Moreover, the study underlines moderating implications of social barriers that will delineate the effectiveness of using different frames (e.g., fear appraisal and safety benefits) of public service message and selection of media (traditional vs. digital) in diminishing the inverse effects of skepticism towards COVID-19.

## 2. Literature Review and Hypotheses Development

### 2.1. The Health Belief Model and Affect Theory

#### 2.1.1. The Perceived Threat and Willingness to Take COVID-19 Vaccine

The HBM was established to underline psychological factors elucidating on the predictors of one’s health-related behaviors [30]. The HBM was initially utilized to understand why tuberculosis prevention programs were not truly effective [31,32]. The HBM was designed to understand why people do not embrace preventive behaviors during health programs [33] and decline to be involved in such campaigns [31]. The original HBM grasps the following four dimensions: perceived susceptibility, perceived severity, perceived benefits, and perceived barriers [34]. Perceived susceptibility theorizes that an intensification in people’s risk perception about a disease will improve their engagement in preventive measures to diminish the perceived risk [33,35]. At the same time, perceived severity encompasses some appraisal of the potential aftermaths of a disease or behavior established on prior health knowledge. It refers to one’s beliefs about the harmful effects of a particular illness that could happen to a person [34]. However, lately, scholars have advocated that two perceived beliefs, (1) susceptibility and (2) severity, can be combined to formulate a single variable, which they refer to as perceived threats [35]. In the current research, one’s beliefs about susceptibility to and the severity of the COVID-19 will be operationalized using a perceived threat. The literature has suggested that the perceived threat is a better predictor than considering both dimensions as a separate variable [35,36].

Furthermore, threat perception has been identified as the focal emotional response, particularly during the epidemics such as transmissible diseases. Adverse emotions after the threat associated with the pandemic can be contagious, and one’s vulnerability to infection can escalate the extent of threat perception. The extant literature affirmed that the intensity of the threat intensifies attention to positive motivation, seemingly to counteract the anxiety associated with a threat (e.g., COVID-19 infection) [25,35,36]. People, therefore, contract and adopt protective behavior to cope with the threat in question. In the context of COVID-19, the perceived threat can produce a substantial positive inclination towards acceptance of the greatest protective behavior [29]. 

The HBM also indicates that the extent of susceptibility and severity amplifies the cognitions about harm and is a fundamental determinant of protective actions [32]. It is safe to argue that people’s perception of threat activates the vulnerability control mechanism whereby people (1) understand they are at a high threat, (2) consider they can efficiently prevent the danger through the adoption of the most protective available mechanism, and (3) thus, become motivated to protect themselves [33]. In this theoretical standard, the study argues that people will be engaged to adopt the COVID-19 vaccine to reduce their vulnerability. In contrast, other protective behaviors such as mask-wearing may be perceived as provisional protective behavior against the threat of COVID-19. Moreover, the COVID-19 vaccine protects against the severity of the disease, while other mitigation behaviors such as mask-wearing and social distancing can only reduce the susceptibility. The HBM asserted that a protective measure is the combined function of both severity and susceptibility [31,32]. Thus, both facets maneuver the individual’s assessment of the threat. Hence, people are expected to appraise and react to the threat of COVID-19 by trying to adopt more protective measures, such as vaccination, to evade the fears and we, therefore, hypothesized the following:

**Hypothesis** **(H1).** *The perceived threat of COVID-19 positively influences the willingness to take a COVID-19 vaccine but more favorably for fear appraisal-framed public service messages compared to those containing safety benefits-framed public service messages.*

#### 2.1.2. The Perceived Benefits and Willingness to Take COVID-19 Vaccine

The next element delineated in the HBM is the perceived benefits. It advocates that people identify the worth and utility of embracing new actions regarding diminishing the threat of disease [37,38]. It follows that if individuals identified their vulnerability to a severe health illness (COVID-19), then they would search for the adoption of the behavior to avoid the health hazard. The perceived belief is one’s belief about the apparent benefit of the obtainable action for diminishing the illness risk (COVID-19). Henceforth, they will perhaps embrace such actions centered on their perceived usefulness in reducing threats. Thus, during the COVID-19 pandemic, people are concerned about their vulnerability to get infected with it. In this scenario, people would pursue the course of action (vaccine, wearing a mask, or social distancing) offered to them. However, research has argued that an individual’s perception development involves a comparison of the functional benefits and usefulness of the adaptive behaviors in question [29]. For example, the functional benefit of COVID-19 vaccine is more advantageous than the continuous social distancing.

It is arguable, therefore, that cognitive evaluation would produce adaptation cues (e.g., vaccine vs. social distancing) [37,38]. Nonetheless, more perceived functional benefits in a preventive behavior undermine a slightly less functional one [30]. Different preventive behaviors against COVID-19 have different benefits associated with them. However, the HBM suggested that individuals are expected to adopt the greatest advantageous preventive behavior to avoid the risk [25]. The HBM proposed multiplicative evaluations of the threat, benefits, and feasibility of preventive behaviors [31]. Thus, individuals process information considering all the factors, and high-risk evasion/high benefit circumstances initiate positive evaluations [28]. Thus, we argue that the benefits associated with the COVID-19 vaccine in terms of safety against threat would be positively evaluated by individuals. Moreover, the literature suggested that the perceived benefits of adopting a particular behavior overshadow the obstacles involved in it; people are expected to adopt a behavior once they trust it will diminish their risks and uncertainties [25,39]. Theoretically, people try to evade risk; thus, the risk of getting a COVID-19 infection will induce more risk perception [40]. The World Health Organization (WHO), the scientific community, and medical practitioners declared that COVID-19 vaccines are safe and ensure protection against COVID-19 infection. Therefore, it is expected that people will perceive vaccination as a course of action that can help them to avoid risk. Contrary to this, COVID-19 vaccine efficacy and safety benefits of the COVID-19 vaccine will provide the course of action to remain safe from getting the infection and we hypothesized the following:

**Hypothesis** **(H2).** *Perceived benefits of the COVID-19 vaccine positively influence willingness to take a COVID-19 vaccine but more favorably for fear appraisal-framed public service messages compared to those containing safety benefits-framed public service messages.*

#### 2.1.3. The Self-Efficacy and Willingness to Take COVID-19 Vaccine

In addition to these dimensions, a self-efficacy construct was included in the model [31]. Similar to the theory of planned behavior’s construct of behavioral control, self-efficacy taps one’s aptitude and competence to adopt novel actions. Self-efficacy refers to a belief that a person can effectively perform the behavior in question despite the perceived barriers. To exemplify this, scholars have noted that people who trust in their capabilities to accomplish, and handiness in accomplishing, a particular objective are more presumable to accomplish that objective [41]. The HBM also suggests self-efficacy as a certainty in prompting well-being and preventive behaviors. It refers to individuals’ belief in their capability to involve safety behavior to yield the anticipated consequence. Principally, self-efficacy implies the following two connotations [29]: (1) apprehensions of people’s certainty that they can vigorously perform the preventive behavior and (2) certainty that the preventive behavior can lead to the anticipated benefits. Consequently, self-efficacy influences how people feel, think, and act regarding risk-taking behaviors (Wong and Yang, 2020).

Research has revealed that when exposed to a higher degree of threat (i.e., severe diseases such as during a pandemic) one is susceptible to instill a higher degree of self-efficacy [35]. For example, one’s belief that adopting precautionary measures such as inoculation can prevent the threat fosters his/her self-efficacy. In brief, the HBM recommends that the association between risk and efficacy is procreative, in such a way that high threat circumstances pledge high self-efficacy [36]. Therefore, in high-risk circumstances such as COVID-19, positive behavioral changes (e.g., the willingness of COVID-19) are expected owing to the activation of the danger control mechanism. The HBM, therefore, explains the interplay between numerous beliefs such as threat, benefits, and self-efficacy that regulate the preventive outcomes [31,32]. Mainly, an individual’s threat perception predicts the amount of response to the given information. For instance, how strongly a preventive response is supported or outlawed [30]. In contrast, self-efficacy predicts the nature of the response, whether a preventive response is feasible or not [28]. In this regard, studies have reported that the COVID-19 pandemic corresponded to a high level of threat among the public [42]. However, these studies have described a low level of self-efficacy mainly due to a lack of safety action cues. The belief to organize, execute, and manage their prospective situation was low (e.g., mask-wearing is not a permanent solution). They were afraid of coping adequately with the situation provided. At the beginning of the pandemic, people sensed more threats to their safety, but the development of the vaccines has provided them with the capability to accomplish safety goals. In this regard, the availability of vaccines has enhanced their belief that immunization is an adequate behavior and, thus, we hypothesized the following:

**Hypothesis** **(H3).** *Self-efficacy positively influences the willingness to take a COVID-19 vaccine but more favorably for fear appraisal-framed public service messages compared to those containing safety benefits-framed public service messages.*

#### 2.1.4. Public Service Message Framing, Media Type, and Willingness to Take COVID-19 Vaccine

In addition to these factors, the HBM also postulates that people’s behavior can also be influenced by cues to actions in conjunction with the four perceptions. Scholars categorized these cues into the following two categories: external and internal cues [29]. External cues are social influences (e.g., peers, media, health experts, etc.) and some peripheral influences that can inform or promote awareness to provoke preventive behaviors [29,35]. On the other hand, internal cues comprise psychosomatic prompts (e.g., discomfort or indications) that prompt one to embrace new behaviors [34]. These internal cues mainly deal with emotional feelings. The present study aims to investigate the utility of the communication campaign predicting the willingness to take the COVID-19 vaccine in Pakistan. Thus, the study integrated the HBM that emphasizes the characteristics associated with cognition and affect theory that emphasize feelings. It has been established in the persuasion literature that people’s decisions are reliant on cognitive and emotional feelings [29].

Previous research has shown that media health campaigns influence individuals’ health behavior [34,41]. Most health campaigns used public service messages and manifested the cognitive and emotional cues to obtain the desired results [41]. This phenomenon of message framing has been widely used in health communication to induce motivational protective behaviors. The framing in public service messages is intended to achieve specific connotations through exhibiting facts (cognitive) or emotion (affect) in messages [39]. Thus, these strategic messages are designed to manipulate the targeted behaviors among the public. The frame selection of these messages is at a par with the strategic needs. For example, during COVID-19 safety benefits, messages can reduce the skepticism related to the vaccine’s side effects.

Similarly, a fear appraisal can work to enhance the extent of threat perception among the public [29]. This research emphasizes investigating the efficacy of cognitive and affective framed messages manifested from safety benefits and threat appraisal messages. Extensive evidence affirmed that public service messages that include facts underlining the safety benefit, such as vaccination health benefits to avoid infection, can prompt preventive behaviors (vaccination). However, the health communication literature could advocate that fear appraisal can reinforce protection behavior more immensely [32]. For instance, a study found that underscoring fears in communication messages can lead individuals to change their behavior more intensely than cognitive ones [43].

**Figure 1:**
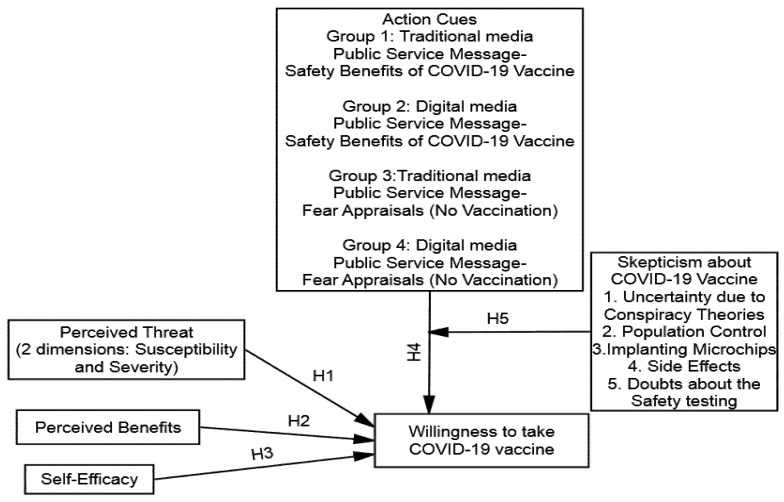
Conceptual Model of Study.

Furthermore, a plethora of research has noted that message source credibility is also crucial to motivate an individual’s careful deliberation [44,45]. If the message’s source is perceived as trustworthy, then there is a greater chance of persuasion [46]. Therefore, during the COVID-19 pandemic, mainly, we assume that due to the spread and impression of misinformation on digital media, people’s willingness to take the COVID-19 vaccine will be influenced more by traditional media sources (see Figure 1). Based on the above premises, it is likely that public service messages manifested with fear appraisals on traditional media will be effective in reducing vaccine hesitancy. We hypothesized that trusted voices had been shown to make public health messages more acceptable.

**Hypothesis** **(H4).** *Traditional media comprising public service messages of fear appraisals not getting COVID-19 vaccines favorably influences willingness to take COVID-19 vaccine compared to other action cues.*

#### 2.1.5. Moderation of Skepticism towards COVID-19 Vaccines

The perceived barriers—the last dimension presented in the HBM—is the utmost prevailing element of the HBM [41,47], it is related to the people’s appraisal of the hindrances and complications they possibly will meet once they embrace a new behavior. However, the perceived barriers could cause one’s halting adoption behavior in question [35,48]. Thus, according to the HBM, people generally appraise new behaviors’ usefulness and probable outcomes before undermining the existing behaviors [30,49]. The COVID-19 pandemic has already seen the rise of an infodemic, characterized by many conspiracy theories that can hinder the efforts to curtail the spread of a virus and mass inoculation [50]. In contrast to traditional media, social media content is created, liked, and shared without editorial oversight. Thus, frequently shared posts on social media platforms generally rely on opinionated content rather than scientific evidence [16]. In this regard, Basch et al. (2017) investigated 87 YouTube videos and found that 65 percent of them articulated anti-vaccine sentiments [17]. Recently, the top YouTube videos about COVID-19 expressed non-factual information about vaccines, and, surprisingly, these videos were watched by more than 60 million viewers [6]. Similarly, it was found that anti-vaccine tweets were re-tweeted more than neutral tweets between 2010 to 2016 [18]. Another study examined 150 Instagram posts and found that anti-vaccine posts have a significantly higher average number of likes [19]. In sum, there is a wide range of conspiracy theories that surround COVID-19, for example, a virus created as a bioweapon [51], the virus is a source to reap profits for pharmaceutical companies [52], COVID-19 is a plan to implant the microchips in the name of vaccination for birth control [53]. Recent studies have also reported that skepticism has an inverse influence on the willingness to take the COVID-19 vaccine and function as a barrier [54]. Therefore, the study proposed the inverse moderating influence of skepticism towards COVID-19 vaccines and hypothesized the following:

**Hypothesis** **(H5).** *The skepticism towards COVID-19 vaccines (barriers) inversely moderates the relationship between media public service messages and the willingness to take a COVID-19 vaccine.*

## 3. Materials and Methods

### 3.1. Design, Participants, and Procedure

This research employed a cross-sectional experimental factorial 2 (type of media: traditional vs. digital media) X 2 (message frame: health and safety benefit of COVID-19 vaccine vs. fear appraisal) between-subject design to examine the effectiveness of the promotional messages in promoting immunization behavior towards COVID-19 vaccine among the people. The experimental design rigorously provides (a) a high level of control, (b) strong internal validity compared to other methods, (c) evidence on the underlying cause, and (d) replication conditions; thus, it can yield findings that are explicit and pertinent with consistency [39,55]. Hence, experiments can be used to recognize a causal association that is somewhat problematic and non-viable to do with other designs [56,57]. Therefore, this study provides vigorous empirical evidence about the effectiveness of the messages’ attributes and sources commonly used in recent COVID-19 vaccination awareness campaigns. To do so, the study employed four conditions to the participants, and they were administrated to have disclosure of distinct manipulation. The study utilized the multistage sampling method, a convenience and random sampling method. In the first stage of the multistage, the sample was recruited using multiple means such as social media announcements, and volunteer participation was requested. Due to movement restrictions, we used the online medium to execute the experimental study instead of the lab design. A total of 320 participants were enlisted and secured with a signed and informed ethical consent form. Later, for executing random assignments to the groups, we generated a list of these participants using their emails. The list of the emails was used for the randomly assigned conditions to the participants. The Microsoft excel random assignment function was used to ensure randomization.

These 320 adult Pakistani nationals were then assigned to four groups (*n* = 80) and subjected to different conditions based on design. The Group 1: Health and Safety Benefit of COVID-19 narrated in public service message published in a local newspaper (see Appendix A); Group 2: Health and Safety Benefit of COVID-19, narrated in a promotional message circulating on social media; Group 3: Fear appraisal related to no COVID-19 vaccine immunization, narrated in public service message published in the local newspaper; Group 4: Fear appraisal related to no COVID-19 vaccine immunization, narrated in a promotional message circulating on social media. Furthermore, there is no consensus over the total observations, some scholars agreed upon the 5 observations per variable, while some delineated 10 observations per variable [55]. However, for the experimental designs, the sample size of each group was 80, which is suitable to perform experiments; the literature recommended a minimum of 30 participants in one group as a statistical threshold [56,57].

### 3.2. Instrumentation

#### 3.2.1. Stimuli Selection Procedure and Manipulation Checks

For this study, four fictitious stimuli campaigns (public service messages) were designed; two public service messages included the health safety benefits of a COVID-19 vaccine and two public service messages, including the fears of not having COVID-19 vaccine immunization. The National language (Urdu) was used in the public service messages (please see Appendix A for the English version). Before data collection, translational and face validity was achieved by sending the stimuli, variable operational definitions, and the questionnaire to field experts, including five academicians and five practitioners, with a request to rate the appropriateness of the stimuli on a 4-point Likert scale. They were also requested to include feedback, and this procedure was repeated after making some suggested adjustments to the stimuli (content). The expert rating was computed to observe the content validity rating (CVR); the literature suggests that, in the case of 10 experts, the acceptable threshold is 0.66; therefore, both the stimuli and the questionnaire after the second round of revisions achieved the acceptable level [58,59]. Before moving on to the data collection, a pilot study was also carried out. In total, 28 students participated in this pilot study. The students were randomly assigned to the four conditions of the experiment. For manipulating the independent variable, the study used three items with a 5-point Likert scale reflecting stimuli features. The respondents were asked to give a response on the semantic differential scale after disclosure to relevant stimuli. The statement of the scale reads as “COVID-19 Vaccine” is: (1) 5 = tremendously safe for health, 1 = not at all safe for health (2) 5 = extremely reliable, 1 = not at all reliable, and (3) the information about the COVID-19 vaccine suggest that it is: 1 = immunization from COVID-19 is highly crucial to avoid severe health issues, 5 = immunization from COVID-19 is not at all crucial for avoiding severe health issues.

#### 3.2.2. Perceived Threat of COVID-19

The perceived threat of COVID-19 was assessed using the following 4-item modified version of the 5-point Likert scale “(5 = strongly agree, 4 = agree, 3 = Neutral, 2 = disagree and 1 = strongly disagree)” from the previous studies [35]: (1) “I will likely get the COVID-19 virus infection if I do not get vaccinated”, (2) “I believe that COVID-19 infection is severe”, (3) “The thought of being infected from COVID-19 scares me”, and (4) “Due to my routines, I will more likely be infected from COVID-19 if I do not perform safety behavior”. 

#### 3.2.3. Self-Efficacy towards COVID-19 Vaccine Immunization

The self-efficacy towards COVID-19 vaccine immunization was assessed using a 3-item modified version of the 5 points Likert scale “(5 = strongly agree, 4 = agree, 3 = Neutral, 2 = disagree and 1 = strongly disagree)” from the previous literature [33]. The scale comprised items (1) “I am certain that I could get a future COVID-19 vaccination”, (2) “For me to have a COVID-19 vaccination would be easy”, and (3) “if I wanted to, I could easily have a COVID-19 vaccination”.

#### 3.2.4. Perceived Benefits of COVID-19 Vaccine

The perceived benefit of the COVID-19 vaccine was assessed using a 2-item modified version of the 5 points Likert scale “(5 = strongly agree, 4 = agree, 3 = Neutral, 2 = disagree and 1 = strongly disagree)” from the previous literature [33]. The scale comprised items (1) “Getting the COVID-19 vaccine will decrease my chances of dying from the COVID-19 infection”, and (2) “Getting the COVID-19 vaccine is the best way for me to avoid severe effects of COIVD-19 infection”.

#### 3.2.5. Skepticism towards COVID-19 Vaccines (Barriers) 

In the present study, the perceived barriers have been operationalized as skepticism towards COVID-19 vaccines. The study has identified five potentials and common skepticisms about the COVID-19 vaccine after an intensive literature review. Thus, it was assessed using a 5-item modified version of the 5-point Likert scale “(5 = strongly agree, 4 = agree, 3 = Neutral, 2 = disagree and 1 = strongly disagree)” from the previous literature [46]. The respondents were requested to give response to the following items: (1) “I am afraid to have a COVID-19 vaccine because I do not understand what will be done”, (2) “I am afraid to have a COVID-19 vaccine because its development was too rushed to test safety”, (3) “I am afraid of getting a COVID-19 vaccine because it is a Population Control Mechanism”, (4) “I am afraid of getting a COVID-19 vaccine because I am concerned about a bad reaction to the vaccine”, and (5) “I am afraid of getting a COVID-19 vaccine because the microchip can be implanted in my body”.

#### 3.2.6. Willingness to Take COVID-19 Vaccine

The willingness to take a COVID-19 vaccine was assessed using the following 3-item modified version of the 5 points Likert scale “(5 = strongly agree, 4 = agree, 3 = Neutral, 2 = disagree and 1 = strongly disagree)” from the previous literature [11,54]: (1) “I am willing to take COVID-19 vaccine”, (2) “I am willing to purchase COVID-19 vaccine, if not available for free in my country”, and (3) “I am willing to any available COVID-19 vaccines without considering the country of origin”.

## 4. Results

### 4.1. Demographic and Preliminary Analysis

We measured the typical demographic characteristics as control variables. Precisely, we measured for the participant’s gender, age group, education level, income status, previous medical history, and marital status. The demographic characteristics analysis has been reported in Table 1. The study has followed the stepwise analytical approach reported in the hypotheses section and reported no potential effect of the demographic variables. Detail is available in the hypotheses testing section.

The preliminary data analysis has been performed statistically on SPSS software. During the descriptive analysis, we evaluated (1) data normality through visual and statical inspection, (2) outliers’ visualization, (3) variance inflation (VIF) assessment for determining any possibility of multiclonality, and (4) Pearson’s test for correlation. The descriptive analysis of all the groups (*n* = 320) has been conducted; that is presented in Table 2. The results demonstrated satisfactory results of normality, bivariate correlations, and found no issue of multicollinearity.

### 4.2. Manipulation Checks 

The study used the post hoc ANOVA *t*-test to confirm the manipulation interventions across the four groups. The findings of the ANOVA suggested significant mean differences; greater mean values were observed in the respondents that were exposed to the public service messages that included fear appraisals (MeanG3 = 4.56, SD = 1.12; MeanG4 = 4.29, SD = 1.06) in contrast to those who were exposed to the public service messages that included safety benefits (MeanG1 = 3.81, SD = 0.89 and MeanG2 = 3.54, SD = 0.74). Therefore, the findings verified the manipulation of the stimuli (t = 6.87; *p* ≤ 0.00). Additionally, Levene’s test of variance was performed to validate these reported differences between the subjects exposed to diverse sorts of public service messages, which also confirmed variances (F (1384) = 43.56, *p* ≤ 0.00).

### 4.3. Confirmatory Factor Analysis (CFA) 

Next, the study employed the structural equation modeling techniques (hereafter SEM) and conducted several confirmatory factor analyses (hereafter CFA) using AMOS 24.0. This technique is suitable to verify the validity and the fitness of the models. Initially, the research used CFA to examine the following: (1) Convergent and discriminant validity, (2) measurement model fittest, and (3) structural model fitness. However, later multi-group CFA was used for hypothesis testing. Primarily, four CFAs were performed based on the hypothesized proposed measurement models.

Each measurement model was derived from the data representing each experimental condition; (Group one) Traditional Media–Public Service Message–Safety Benefits (Group two) Health Digital Media–Public Service Message–Safety Benefits (Group three) Traditional Media–Public Service Message–Fear Appraisals, and (Group four) Digital Media–Public Service Message–Fear Appraisals. The four CFAs were carried out by loading the items on the parent variable to evaluate the validity and model fitness using suggested thresholds of the indices [60]. The model fit indices have been reported in Table 3, obtained after the deletion of only one item from the SV variable from each model. 

Scholars noted that the chi-square per degree of freedom and absolute measures (e.g., GFI and RMSEA) are sensitive to the sample size, while incremental fit indices are less sensitive to the sample size [61]. The study employed incremental fit indices such as TLI, IFI, and CFI, which demonstrate to be more apt indicators under smaller sample size conditions. However, we also used the chi-square per degree of freedom, absolute measures (e.g., GFI and RMSEA), and incremental fit indices (e.g., TLI, IFI, and CFI) to verify the model fitness. The study used multiple (e.g., six) indices to crosscheck the validity of the measurement model fitness outcomes.

Furthermore, the convergent validity was examined using the recommended values of the composite reliability (more significant than 080) and the average variance extracted (greater than 0.50). The research employed the Fornell and Larcker method to calculate the discriminant validities of the variables involved in this study. The results indicated that discriminant validity had been proven across all the experimental groups because the latent variables accounted for more variation in their associated parent variables than it shared with other variables in a similar model (see Table 4). Before proceeding to the hypothesis analysis, the structural models for each group were examined for the model fitness and revealed satisfactory results across all four experimental conditions. These data are accessible in Table 3.

### 4.4. Hypothesis Testing

**Figure 2:**
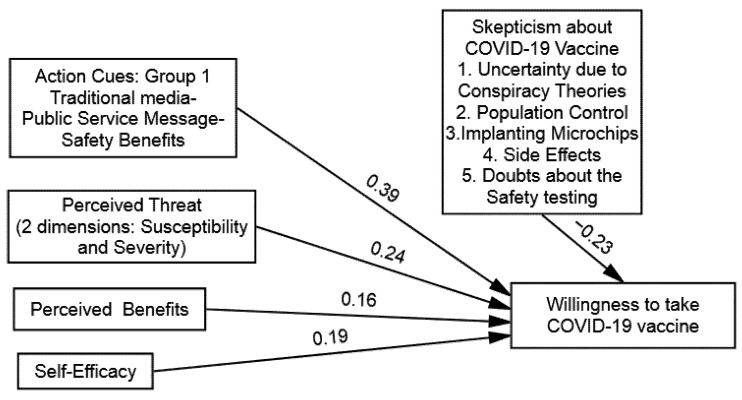
Structural model of group 1: Traditional Media–Public Service Message–Safety Benefits.

**Figure 3:**
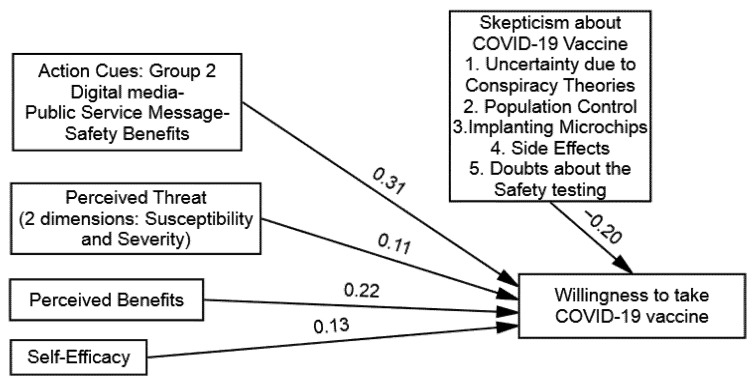
Structural model of group 2: Digital Media–Public Service Message–Safety Benefits.

**Figure 4:**
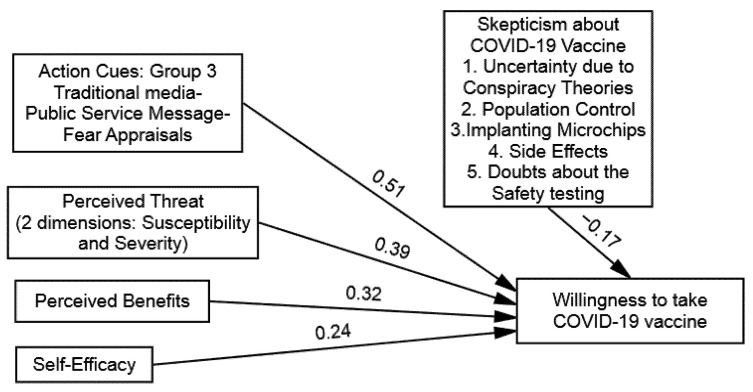
Structural model of group 3: Traditional Media–Public Service Message–Fear Appraisals.

**Figure 5:**
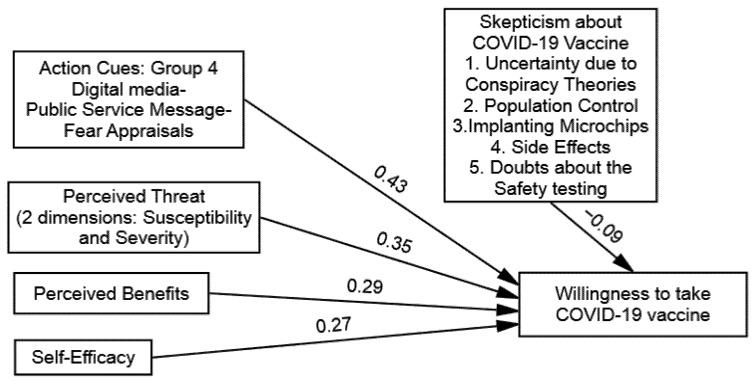
Structural model of group 4: Digital Media–Public Service Message–Fear Appraisals.

This research employed the multi-group confirmatory factor analysis (hereafter MG-CFA) method. The MG-CFA method is essential and recommended to confirm the presence of measurement invariances across the groups. Therefore, the measurement model has been derived from the data obtained from four experimental conditions. The multi-group model includes data of 320 subjects in total, and four groups were computed. To verify the measurement invariances, three parameters were used to evaluate the following: (1) the MG-CFA model with constrained paths, (2) the MG-CFA model with constrained paths, and (3) CFI difference of 0.01. The outcomes illustrated that all the conditions had significant variances constrained in all paths as x^2^ (difference) = 2.89, the degree of freedom (difference) = 4, and *p* = 0.001, thus, the differences were found to be significant. Similarly, the outcomes of unconstrained paths were also revealed as significant, as x^2^ (difference) = 3.23, the degree of freedom (difference) = 4, and *p* = 0.001. The CFI differences were also below the threshold of 0.01, which revealed that there is no issue of the measurement invariance. Henceforth, the research continued for the inferential statistics (see Table 5 and Figure 2, Figure 3, Figure 4 and Figure 5). 

For the hypothesis testing, the study used the phase-wise analytical technique and added the preliminary variables involved in the study. After adding them, control variables were also introduced in the model to observe any potential influence of control variables. However, the analysis revealed no significant influence of the control variables, as no variance change (R^2^) had been observed after entering them into the model. The study proposed four hypotheses postulating the direct influences of (H1) the perceived threats of COVID-19, (H2) perceived benefits of a COVID-19 vaccine, (H3) self-efficacy, and (H4) public service messages about a COVID-19 vaccine on the willingness to take a COVID-19 vaccine with varying strengths across the four conditions. Therefore, multi-group (SEM) was used to find and compare the strength of these proposed influences. 

The findings of multi-group (SEM) demonstrated that the direct influence of the perceived threats of COVID-19 on the WTV was significant across all the groups, as follows: (1) group one (β = 0.24 and *p* = 0.01), (2) group two (β = 0.11 and *p* = 0.01), (3) group three (β = 0.39 and *p* = 0.01), and (4) group four (β = 0.35 and *p* = 0.01). Hence, (H1) was supported in that perceived threats of COVID-19 will influence the WTV among the participants exposed to the public service messages, including fear appraisals (see Table 5). Likewise, the findings suggested that the direct influence of the perceived benefits of the COVID-19 vaccine on the WTV was significant across all the groups, as follows: (1) group one (β = 0.16 and *p* = 0.05), (2) group two (β = 0.22 and *p* = 0.01), (3) group three (β = 0.32 and *p* = 0.01), and (4) group four (β = 0.29 and *p* = 0.01). Hence, (H2) was also supported, given that the perceived benefits of a COVID-19 vaccine will have more influence on the WTV among the participants exposed to the public service messages that included fear appraisals (see Table 5 and Figure 2, Figure 3, Figure 4 and Figure 5). 

Further, the findings of the multi-group (SEM) demonstrated that the direct influence of the self-efficacy to get the COVID-19 vaccine on the WTV was also significant in all the groups, as follows: (1) group one (β = 0.19 and *p* = 0.01), (2) group two (β = 0.13 and *p* = 0.01), (3) group three (β = 0.24 and *p* = 0.05), and (4) group four (β = 0.27 and *p* = 0.01). Hence, (H3) also supported that self-efficacy will have more influence on the WTV among the participants exposed to the public service messages, including fear appraisals (see Table 5). Lastly, the direct influence of the public service messages about the COVID-19 vaccine on the WTV was also found significantly across all the groups, as follows: (1) group one (β = 0.39 and *p* = 0.01), (2) group two (β = 0.31 and *p* = 0.01), (3) group three (β = 0.51 and *p* = 0.01), and (4) group four (β = 0.43 and *p* = 0.01). Hence, (H4) was also supported, as public service messages about the COVID-19 vaccine will influence the WTV among the participants exposed to the public service messages, including fear appraisals (see Table 5).

### 4.5. Moderation Analysis

Onwards for the moderation hypothesis testing (H5) postulated skepticism towards COVID-19 vaccines (barriers) as the moderating factor in determining the relationship between the public service messages about COVID-19 and the WTV across the four experimental groups. The research used the recommended hierarchal linear modeling (HLM) method along with employing bootstrapping procedures by conducting multi-group analysis to compare the moderation strength. To do so, four models were computed (one for each group). The main influence of public service messages about the COVID-19 vaccine on the WTV was also found significant across all the groups, as follows: (1) group one (β = 0.39), (2) group two (β = 0.31), (3) group three (β = 0.51), and (4) group four (β = 0.43). Distinct models were computed to identify the instructional effectiveness of the public service message and skepticism towards COVID-19 vaccines. The direct effect of skepticism towards COVID-19 vaccines on the WTV was found to be negative and significant in all the conditions, as follows: (1) group one (β = −0. 23), (2) group two (β = −0. 20), (3) group three (β = −0. 17), and (4) group four (β = −0. 09). After calculating the main effects of PSM and SV, we added the interaction term of (PSM X SV) to explore the proposed inverse moderation of the SV in the relationship between the PSM and the WTV that was found to be significant in all conditions, as follows: (1) group one (β = −0. 14), (2) group two (β = −0. 26), (3) group three (β = −0. 09), and (4) group four (β = −0. 11). The findings offered in Table 6 clarified that the strength of the PSM and the WTV is an inverse function of the skepticisms towards COVID-19 vaccines and supported (H5). 

## 5. Discussion

### 5.1. Vaccine Willingness 

This research used cross-sectional experimental factorial 2 (type of media: traditional vs. digital media) X 2 (message frame: health and safety benefit of COVID-19 vaccine vs. fear) between-subject design to examine the effectiveness of promotional messages concerning immunization behavior towards a COVID-19 vaccine among the general public in Pakistan. The study posed five hypotheses. The findings of this study supported the four hypotheses, whereas hypothesis five found an inverse effect regarding the willingness to take COVID-19 vaccine. The findings of hypothesis one illustrate that the perceived threat of COVID-19 positively influences the willingness to take a COVID-19 vaccine more favorably for fear appraisal-framed public service messages than safety benefits framed public service messages. These findings are similar to the previous literature that found that participants who have a higher perceived severity of COVID-19 infection, perceived susceptibility, and perceived benefits of COVID-19 vaccination were more willing to take the COVID-19 vaccine [23]. Threat appraisal corresponds to an increase in vaccine willingness [9]. Likewise, the results of H2 and H3 suggested that the perceived benefits of COVID-19 vaccines and self-efficacy were also found to positively influence the willingness to take the COVID-19 vaccine more favorably for fear appraisal-framed public service messages as compared to safety benefits public service messages. Similarly, our findings elucidated that self-efficacy positively influences the willingness to take a COVID-19 vaccine more favorably for fear appraisal-framed public service messages than those containing safety benefits. Furthermore, the findings of H5 suggested that when individuals have a higher level of skepticism toward COVID-19 vaccines, they would have a lesser willingness to take COVID-19 vaccines. However, the overall variance extracted in the models remains positive in all conditions. These findings give a fascinating insight into the strength of the public service messages in promoting a willingness to take COVID-19 vaccines among the general public. Albeit certain skepticisms towards COVID-19 vaccines have an inverse role, public service messages, regardless of the medium, positively influence one’s willingness to take COVID-19 vaccines. Concerning the role of action cues, we found that the traditional media’s public service message of fear appraisals of not getting COVID-19 vaccine influence the willingness to take a COVID-19 vaccine more favorably than other action cues.

### 5.2. Trust in Vaccines

Public trust in vaccines is another global issue. To increase the confidence in the COVID-19 vaccine, there is a dire need to address the false narratives on social media platforms [26]. During a time of crisis such as the COVID-19, the public reliance on media increases many-fold. However, media, especially social media, is prone to un-regulated, misinformation, non-factual information, opinionated, and anti-vaccine content [6,16,17,59]. This kind of content is one of the major reasons for generating and sustaining vaccine hesitancy among the public [60]. The findings of H5 explicate that the misinformation and conspiracy theories widely circulated on social media platforms make it more challenging for public health officials to inform and convince the public about vaccine benefits. In this regard, the proper editorial gatekeeping of science communication via publishing, broadcasting, and sharing factual and scientific information about the COVID-19 vaccines can increase public trust in vaccines. 

### 5.3. Vaccine Hesitancy

The public’s vaccine hesitancy can be decreased by providing information on the safety of the COVID-19 vaccine. Moreover, it is noted that to increase willingness and minimize hesitancy among the public to get vaccinated is to publicize the social benefits of the vaccines [24]. Interestingly, the findings of this study elucidate that the skepticisms towards COVID-19 vaccines (barriers) inversely moderates the influence of media public service messages and willingness to take COVID-19 vaccines. The hesitancy to take the COVID-19 vaccine is associated with conspiracy theories. This finding also supports the previous studies that found that conspiracy theories influence people’s intention to get vaccinated [62,63]. These conspiracy theories revolve around vaccine side effects and population control through the COVID-19 vaccine [7,26]. The previous research shows that civic dialogue with vaccine-hesitant people is a significant step to address their concerns about vaccine efficacy [64,65]. The hesitancy to get vaccinated can be minimized through these means, and individuals could be convinced to be inoculated. Importantly, our findings of H4 and H5 suggest that willingness to get the COVID-19 vaccine is influenced by media, public service messages, especially fear appraisal-framed public service messages compared to safety benefits public service messages. Thus, the media can be used effectively to reduce the hesitancy of the vaccine refusal groups. Moreover, the content shared on social media platforms can be moderated to make it more vaccines friendly and factual, preferably sharing scientific evidence instead of sharing non-factual information. 

### 5.4. Managerial Implications

These findings provide greater insight for the policymakers to respond to this emergency of vaccine hesitancy. The study gives timely information about the strategic utilization of communication resources. Past theories suggested that when apparent barriers and benefits have been recognized, then the strategic remedy of the issue can be carried out by providing action cues to the public. In the context of COVID-19, the development of vaccines has been established as a beneficial product, while vaccine hesitancy has been identified as a barrier. Thus, the result of this study in response provides valuable strategic information about the selection of the fear appraisal public service messages through the traditional and digital media. The experimental results suggestions can be utilized in upcoming government campaigns in Pakistan to address the vaccine hesitancy issue. Moreover, public service messages about the COVID-19 vaccine on the traditional media were remained more effective (G1_safety_β = 0.39, (G3_fear_β = 0.51) compared to the digital media (G2_safety_β = 0.31, (G4_fear_β = 0.43) in predicting the WTV. The policy makers, thus, may disseminate the public service messages about the COVID-19 vaccine on traditional media more recurrently.

### 5.5. Limitations and Future Research

Though this experimental study has made timely and appropriate contributions to the body of knowledge to address the issue of vaccine hesitancy, there are, however, numerous restrictions. First, because of the movement restrictions, the study utilized the online data collection procedure that may influence the internal validity of these results. In normal time, lab design is advocated for use. Although we endeavored to ensure randomization through generating the list of these participants’ emails, nonetheless, the experimental study was administered online. Second, the data were collected only from internet users. Future studies can conduct field surveys to enhance the generalization of the results by using more sophisticated sampling selection methods. Third, the study employed the convenience sampling method; thereby, it is not plausible (a) to estimate a survey response rate, (b) to indicate the confidence limits of the findings, and (c) to clarify what subgroup of the population the survey respondents represent. Fourth, although this study manipulated COVID-19 vaccination awareness by using fear and safety recommendations as public services messages, the other message attributes (e.g., message format and source credibility) could not be rigorously controlled. In future research, probable confounding variables, such as earlier exposure and personnel attributes, are likely to better tap the effects of message attributes. Finally, this study was conducted in Pakistan, therefore, the findings of the study can be generalized to other countries and contexts with caution.

## 6. Conclusions

The development of the COVID-19 vaccines has been a great breakthrough in recent human history in eradicating the threats of the virus to human life. Though the immunization has been confirmed to be harmless for public health and efficacious against COVID-19, regardless of its benefits, public reluctance in taking the vaccine because of the widespread skepticism is challenging vaccination campaigns. In this scenario, we have provided a theoretical-driven clarification about the role of communication messages and medium selection to support vaccination campaigns. Therefore, the experimental study tested the four COVID-19 vaccination communication campaigns using different messages inoculation techniques across traditional and digital media. The results of the series of four experimental studies found that fear appraisal messages can inoculate strong effects to diminish skepticism. Therefore, we suggest that fear appraisal messages persuading people to get early vaccines can be a useful strategy before it is too late. These strategies are usually incorporated in sales promotion advertisements. Given that fear appeals delineating vulnerability to COVID-19 manifested with an elucidation of timely vaccination to avoid risk is the effective strategy to diminish the hesitancy. We suggest to Pakistani policy makers that the usage of public service messages incorporating such fear appraisals through traditional media can be a more effective strategy to counter vaccine hesitancy.

## Figures and Tables

**Table 1:** Demographic Characteristics.

Demographic	Frequency	Percentage
Gender		
Male	179	55.9
Female	141	44.1
Medical History		
Yes	63	19.7
No	257	80.3
Marital Status		
Yes	177	55.3
No	143	44.7
Age		
18–29	68	21.2
30–44	127	39.7
45–59	94	29.4
60 and above	31	9.7
Education level		
High School Certificate	77	24.1
College/Diploma	152	47.5
University Degree	91	28.4

**Table 2:** Correlations.

G 1	Mean	PT	PB	SE	PSM	SV	WTV	G 2	Mean	PT	PB	SE	PSM	SV	WTV
PT	3.25	1						PT	2.89	1					
PB	3.65	0.41 *	1					PB	3.19	0.37 *	1				
SE	3.56	0.56 *	0.37 *	1				SE	3.72	0.43 *	0.47 *	1			
PSM	3.81	0.37 *	0.39 *	0.40 *	1			PSM	3.54	0.29 *	0.37 *	0.44 *	1		
SV	2.78	−0.31 *	−0.56 *	−0.38 *	−0.27 *	1		SV	2.96	−0.16 *	−0.13 *	−0.22	−0.18 *	1	
WTV	4.09	0.35 *	0.33 *	0.19 *	0.39 *	−0.12	1	WTV	3.89	0.25 *	0.32 *	0.19 *	0.17 *	−0.34 *	1
G 3	Mean	PT	PB	SE	PSM	SV	WTV	G 4	Mean	PT	PB	SE	PSM	SV	WTV
PT	4.57	1						PT	4.13	1					
PB	4.48	0.39 *	1					PB	4.23	0.28 *	1				
SE	4.35	0.43 *	0.37 *	1				SE	3.98	0.31 *	0.65 *	1			
PSM	4.56	0.62 *	0.48 *	0.44 *	1			PSM	4.29	0.36 *	0.76 *	0.65 *	1		
SV	2.38	−0.47 *	−0.26 *	−0.34	−0.43 *	1		SV	2.60	−0.08	−0.24 *	−0.20 *	−0.14 *	1	
WTV	4.43	0.29 *	0.38 *	0.25 *	0.57 *	−0.27	1	WTV	4.28	0.38 *	0.23 *	0.38 *	0.27 *	−0.09	1

G = Group samples from each group = 80, *: *p* =< 0.05. PT = Perceived Threat, PB = Perceived Benefits, SE = Self-efficacy, PSM = Public Service Message, SV = Skepticism towards COVID-19 Vaccines, and WTV = Willingness to take COVID-19 Vaccine.

**Table 3:** Confirmatory Factor Analysis.

Measurement Models	x^2^	x^2^/df	GFI	TLI	IFI	CFI	RMSEA
Group 1: Traditional Media–Public Service Message–Safety Benefits	2379	3.56	0.97	0.93	0.93	0.96	0.042
Group 2: Digital Media–Public Service Message–Safety Benefits	1822	2.67	0.93	0.92	0.91	0.93	0.045
Group 3: Traditional Media–Public Service Message–Fear Appraisals	1547	1.97	0.95	0.98	0.96	0.98	0.037
Group 4: Digital Media–Public Service Message–Fear Appraisals	1169	3.34	0.94	0.97	0.94	0.91	0.032
Structural Models		x^2^/DF	GFI	TLI	IF	CFI	RMS
Group 1: Traditional Media–Public Service Message–Safety Benefits	1052	3.79	0.91	0.96	0.98	0.94	0.051
Group 2: Digital Media–Public Service Message–Safety Benefits	867	3.18	0.94	0.91	0.96	0.95	0.045
Group 3: Traditional Media–Public Service Message–Fear Appraisals	1493	2.15	0.96	0.90	0.93	0.99	0.033
Group 4: Digital Media–Public Service Message–Fear Appraisals	1743	3.43	0.98	0.97	0.95	0.90	0.041

Note: x^2^ = Chi-square df = Degree of Freedom, GFI = Goodness of Fit Index, TLI = Turkey Lewis Index, CFI = Comparative fit index, IFI = Incremental fit index, and RMSEA = Root mean square error of approximation.

**Table 4:** Validity Statistics and Standardized Weights.

Items	Group 1: Traditional Media–Public Service Message–Safety Benefits				Group 2: Digital Media–Public Service Message–Safety Benefits				Group 3: Traditional Media–Public Service Message–Fear Appraisals				Group 4: Digital Media–Public Service Message–Fear Appraisals			
	α	CR	AVE	L	α	CR	AVE	W	α	CR	AVE	L	α	CR	AVE	L
PT1	0.88	0.91	0.73	0.86	0.82	0.90	0.70	0.91	0.94	0.94	0.79	0.88	0.78	0.82	0.61	0.71
PT2				0.84				0.78				0.94				0.87
PT3				0.95				0.79				0.83				0.75
PT4				0.78				0.87				0.90				0.38 *
PB1	0.85	0.80	0.65	0.74	0.93	0.90	0.81	0.96	0.77	0.81	0.67	0.78	0.87	0.85	0.74	0.87
PB2				0.89				0.84				0.86				0.85
SE1	0.83	0.88	0.70	0.86	0.78	0.85	0.66	0.89	0.75	0.84	0.64	0.78	0.92	0.91	0.76	0.93
SE2				0.76				0.82				0.93				0.82
SE3				0.89				0.71				0.68				0.87
PSM1	0.89	0.90	0.74	0.87	0.93	0.92	0.77	0.85	0.84	0.88	0.73	0.95	0.76	0.83	0.62	0.76
PSM2				0.78				0.92				0.87				0.82
PSM3				0.93				0.87				0.73				0.79
SV1	0.90	0.92	0.69	0.84	0.84	0.88	0.68	0.78	0.89	0.94	0.74	0.90	0.82	0.89	0.63	0.76
SV2				0.89				0.86				0.91				0.83
SV3				0.91				0.95				0.87				0.92
SV4				0.77				0.69				0.73				0.75
SV5				0.73				0.32 *				0.88				0.68
WTV1	0.86	0.89	0.72	0.90	0.73	0.85	0.65	0.76	0.91	0.91	0.77	0.93	0.84	0.86	0.67	0.88
WTV2				0.84				0.79				0.89				0.81
WTV3				0.81				0.87				0.81				0.76

PT = Perceived Threat, PB = Perceived Benefits, SE = Self-efficacy, PSM = Public Service Message, SV = Skepticism towards COVID-19 Vaccines, and WTV = Willingness to take COVID-19 Vaccine, L = item loadings, CR = Composite Reliability, AVE = Average Variance Extracted, and *: =removed items.

**Table 5:** Hypothesis Testing.

Direct Influence	PT→WTV (H1)	PB→WTV (H2)	SE→WTV (H3)	PSM→WTV (H4)
Group 1: Traditional Media–Public Service Message–Safety Benefits	0.24 *	0.16 *	0.19 *	0.39 *
Group 2: Digital Media–Public Service Message–Safety Benefits	0.11 *	0.22 *	0.13 *	0.31 *
Group 3: Traditional Media–Public Service Message–Fear Appraisals	0.39 *	0.32 *	0.24 *	0.51 *
Group 4: Digital Media–Public Service Message–Fear Appraisals	0.35 *	0.29 *	0.27 *	0.43 *

*: *p* = ≤ 0.05.

**Table 6:** Moderation Results.

Stepwise Moderation	Results
Group 1: Traditional Media–Public Service Message–Safety Benefits, Dependent Variables: WTV	
Step 1: Independent Variables: Public Service Message	0.39 * (5.21)
Skepticisms towards COVID-19 Vaccines	−0.23 * (2.34)
R^2^ Step 2: Moderator: Public Service Message X Skepticism towards COVID-19 Vaccines	0.57
	−0.14 * (3.56)
R^2^	0.47
ΔR2	−0.10
Group 2: Digital Media–Public Service Message–Safety Benefits, Dependent Variables: WTV	
Step 1: Independent Variables: Public Service Message	0.31 * (4.79)
Skepticisms towards COVID-19 Vaccines	−0.20 * (7.35)
R^2^ Step 2: Moderator: Public Service Message X Skepticism towards COVID-19 Vaccines	0.41
	−0.26 * (5.63)
R^2^	0.32
ΔR2	−0.08
Group 3: Traditional Media–Public Service Message–Fear Appraisals, Dependent Variables: WTV	
Step 1: Independent Variables: Public Service Message	0.51 * (4.37)
Skepticisms towards COVID-19 Vaccines	−0.17 * (6.59)
R^2^ Step 2: Moderator: Public Service Message X Skepticism towards COVID-19 Vaccines	0.71
	−0.09 * (6.27)
R^2^	0.65
ΔR2	−0.06
Group 4: Digital Media–Public Service Message–Fear Appraisals, Dependent Variables: WTV	
Step 1: Independent Variables: Public Service Message	0.43 * (3.68)
Skepticisms towards COVID-19 Vaccines	−0.09 * (7.19)
R^2^ Step 2: Moderator: Public Service Message X Skepticism towards COVID-19 Vaccines	0.61
	−0.11 * (9.26)
R^2^	0.57
ΔR2	−0.04

Note. The values in parentheses represent t statistics. Entries are random effects with a robust standard error. R^2^ = proportion of variance explained by antecedent in both models 1 and 2, *: *p* =< 0.05.

## Data Availability

The data that support the findings of this study are available from the corresponding author upon reasonable request due to ethical and privacy restrictions.

## Appendix A

### Appendix A.1. Content of the Message for Group 1: Traditional Media–Public Service Message–Safety Benefits

Narrated in a public service message published in a newspaper with experts, the WHO’s organizational logo, and medical equipment in the display: The World Health organization, scientific community, and medical practitioners declared that COVID-19 vaccines are safe and ensure protection against COVID-19 infection. Therefore, scientists who have researched COVID-19 vaccine efficacy and safety agree with the position that “all COVID-19 vaccines currently available are safe for human health”. COVID-19 vaccines are beneficial for our human health. Hence, vaccination can help to combat the COVID-19 pandemic and ensures global public health.

### Appendix A.2. Content of the Message for Group 2: Digital Media–Public Service Message–Safety Benefits

Narrated in a public service message published online on a digital platform with experts, the WHO’s organizational logo, and medical equipment in the display: The World Health organization, scientific community, and medical practitioners declared that COVID-19 vaccines are safe and ensure protection against COVID-19 infection. Therefore, scientists who have researched COVID-19 vaccine efficacy and safety agree with the position that “all COVID-19 vaccines currently available are safe for human health”. COVID-19 vaccines are beneficial for our human health. Hence, vaccination can help to combat the COVID-19 pandemic and ensures global public health.

### Appendix A.3. Content of the Message for Group 3: Traditional Media–Public Service Message–Fear Appraisals

Narrated in a public service message published in a newspaper with experts, the WHO’s organizational logo, and medical equipment in the display: The COVID-19 pandemic is spreading sharply, wear a mask, and get your vaccines once available before it is too late. The World Health organization, the scientific community, and medical practitioners declared that COVID-19 vaccines could protect against deadly COVID-19 infection. Therefore, scientists who have researched COVID-19 vaccine efficacy and safety agree with the position that “all the COVID-19 vaccines currently available are the last hope for the safety of human health”. COVID-19 vaccines are evading the risk involved in getting infected that may risk ones’ life. Hence, vaccination can help to combat the COVID-19 pandemic and ensures global public health.

### Appendix A.4. Content of the Message for Group 4: Digital Media–Public Service Message–Fear Appraisals

Narrated in a public service message published online on a digital platform with experts, the WHO’s organizational logo, and medical equipment in the display: the COVID-19 pandemic is spreading sharply, wear a mask, and get your vaccines once available before it is too late. The World Health organization, the scientific community, and medical practitioners declared that COVID-19 vaccines could protect against deadly COVID-19 infection. Therefore, scientists who have researched COVID-19 vaccine efficacy and safety agree with the position that “all the COVID-19 vaccines currently available are the last hope for the safety of human health”. COVID-19 vaccines are evading the risk involved in getting infected that may risk ones’ life. Hence, vaccination can help to combat the COVID-19 pandemic and ensures global public health.
